# Neural correlates and neural computations in posterior parietal cortex during perceptual decision-making

**DOI:** 10.3389/fnint.2012.00086

**Published:** 2012-10-10

**Authors:** Alexander C. Huk, Miriam L. R. Meister

**Affiliations:** Center for Perceptual Systems, Institute for Neuroscience, Neurobiology, and Psychology, The University of Texas at AustinAustin, TX, USA

**Keywords:** LIP, posterior parietal cortex, decision-making, neurophysiology, neural correlates

## Abstract

A recent line of work has found remarkable success in relating perceptual decision-making and the spiking activity in the macaque lateral intraparietal area (LIP). In this review, we focus on questions about the neural *computations* in LIP that are not answered by demonstrations of neural *correlates* of psychological processes. We highlight three areas of limitations in our current understanding of the precise neural computations that might underlie neural correlates of decisions: (1) *empirical questions* not yet answered by existing data; (2) *implementation issues* related to how neural circuits could actually implement the mechanisms suggested by both extracellular neurophysiology and psychophysics; and (3) *ecological constraints* related to the use of well-controlled laboratory tasks and whether they provide an accurate window on sensorimotor computation. These issues motivate the adoption of a more general “encoding-decoding framework” that will be fruitful for more detailed contemplation of how neural computations in LIP relate to the formation of perceptual decisions.

## Introduction

“It is an hypothesis that the sun will rise tomorrow: and this means that we do not know whether it will rise.”

—L. Wittgenstein

Some tests of hypotheses are more exciting than others. When measuring neural signals in the lateral intraparietal cortex (LIP) of monkeys while they perform decision-making tasks, it is no longer particularly exciting to observe a correlation between the aggregate spike rate in LIP and the formation of decisions over time. This attitude reflects remarkable recent progress: over the last decade and a half, there have been a large number of demonstrations of LIP activity mirroring the inferred processes of accumulating evidence for the purposes of making a decision during performance of a moving-dot direction discrimination task (reviewed below; also see Gold and Shadlen, 2007).

In this article, we focus on the moving dots paradigm as a specific arena for exploring what such demonstrations of neural correlates tell us about LIP, in part because of our personal familiarity with the details, and in part because the level of detail in this body of work makes for a particularly fruitful discussion. However, this discussion aims for traction with readers who are not yet experts in the dots task paradigm, so we begin by briefly summarizing some of the key results and describing the neural correlate framework. We attempt not just to celebrate the successes of this approach, but to focus scrutiny on what we have not yet learned about LIP function from it. We argue that we know very little about what LIP responses are driven by, how LIP neurons transform their signals into outputs, and what these outputs mean. We propose that this arises from a growing emphasis on neural correlates of psychological processes, over a focus on neural computations of the sort that guides most work in sensory and motor systems. In short, the observation of a neural correlate does not necessarily reveal neural computations. Our goal here is to highlight this distinction, and then attempt to lay groundwork for an increased emphasis on neural computations in posterior parietal cortex.

We conclude that using a more general “encoding-decoding framework” will aid us in unpacking the neural computations in LIP during perceptual decision-making. This framework, which has already proven successful in the study of sensory and motor function, has perhaps even greater potential for unpacking many mechanistic questions about how LIP comes to represent neural correlates of decision variables. Success in this endeavor would also support a more detailed integration of results across the broader literature on LIP function, which contains a variety of experimental paradigms focused on attention, motor intention, visual search, reward expectation, and/or categorization (e.g., Gnadt and Andersen, 1988; Platt and Glimcher, 1999; Bisley and Goldberg, 2003; Dorris and Glimcher, 2004; Sugrue et al., 2004; Freedman and Assad, 2006; Ipata et al., 2006; Thomas and Paré, 2007).

### Basic LIP responses

The longstanding approach for characterizing the basic sensorimotor properties of LIP neurons starts with a simple, instructed eye movement task. When a saccade target is presented in the response field (RF) on an LIP neuron, it usually elicits a brisk visual response. When the subject (a trained rhesus monkey) eventually makes a saccade to that target, the eye movement is complemented by a response burst as well. Thus, the same neuron can exhibit both sensory and (oculo-) motor responses. Furthermore, many LIP neurons also show a persistent, elevated firing rate across the temporal delay between these two events— even when the saccade target is only flashed quickly, and the monkey is required to wait many hundreds of milliseconds before making a saccade to the remembered target location (Gnadt and Andersen, 1988). This persistent activity looks like an explicit neural correlate of the working memory process required in this simple sensorimotor task.

Because the persistent activity of these neurons appeared to explicitly bridge the temporal gap between sensory input and motor output, such cells were theorized to be windows into simple forms of higher cognition (Shadlen and Gold, 2004). Persistent activity allows a neuron's response to be temporally dissociated from the immediate time scales of sensory and motor events, which is likely a key element in the generation of well-considered and temporally-appropriate behaviors in response to prior events (Mountcastle et al., 1975; Fuster, 1997). Furthermore, LIP activity is less tightly related to the occurrence and metrics of saccades, especially compared to related oculomotor areas (Shibutani et al., 1984). Given these putatively “cognitive” response characteristics, LIP is often targeted in neurophysiological investigations of simple cognitive tasks. Although many interesting tasks have been used to probe LIP, here we focus on a particular paradigm that has focused on relating LIP activity to the formation of decisions. This emphasis allows us to discuss detailed neural correlates and neural computations, but the points to be drawn from this exercise are hopefully more general.

### The basic “dots task”

A moving-dot direction-discrimination task (hereafter called the “dots task”) (e.g., Newsome and Paré, 1988) has been frequently used to investigate decision-related signals in area LIP. In this task, the experimental subject performs forced-choice direction discrimination on a random dot kinetogram of variable signal strength. Coherent motion is generated by displacing some proportion of the dots with a spatiotemporal step that yields visual motion. The remainders of the dots are simply replotted in random locations and serve as noise, resembling analog TV snow. The fraction of signal dots is called the “motion coherence,” and serves as a simple way to manipulate the signal-to-noise ratio of motion. The resulting motion is very obvious if the coherence is high or very subtle if the coherence is low. A zero coherence stimulus, which is not readily discernible from a low nonzero coherence stimulus, serves as an elegant means for relating neural to behavioral variability on a single trial level (Parker and Newsome, 1998).

The moving dot stimulus has many important psychophysical properties. First, it yields well-behaved psychometric functions, with a gradual transition from chance to perfect accuracy as coherence is increased. It should also be noted that the signal dots (those chosen to move coherently) are selected anew at random from video update to update. This means that any particular dot is unlikely to continue along a coherent-motion trajectory for a significant amount of time; a signal dot at one point is likely to become a noise dot later, and vice versa. This stochastic nature of the stimulus is likely advantageous: it requires subjects to broadly integrate the net motion over space instead of trying to track a single signal dot; also, it contains a degree of “spatiotemporal splatter” that invites subjects to integrate the directional signals over time. A relatively long psychophysical temporal integration period allows neurophysiologists a longer time period to consider neural responses during a gradual formation of decisions.

This type of random dot kinetogram was originally used by psychologists as a careful stimulus for studying the perception of visual motion (e.g., Anstis, 1970), but the psychophysical components of the dots task proved critical for seminal studies that investigated the relation between the neural activity of middle temporal visual area (MT) and perceptual decisions (e.g., Newsome et al., 1989; Britten et al., 1992). The dots task then evolved into a well-controlled experimental paradigm for studying LIP signals while monkeys decided which direction of motion was presented, and communicated their choice with an eye movement to one of two choice targets located inside and outside of the LIP neuron's RF (Shadlen and Newsome, 1996). Just as the early investigations of LIP focused on visually-instructed saccades to the RF, these later studies focused on visually-informed decisions to make a saccade either to the RF or to another location.

Use of the dots task for studying decision signals in LIP was enriched by the fact that the earlier studies using the same paradigm had quantitatively characterized the responses of MT neurons to these stimuli (Newsome et al., 1989; Britten et al., 1992, 1993), and had also compellingly demonstrated that these MT signals were used by the monkeys in performing the task (Newsome and Paré, 1988; Britten et al., 1996). In short, MT neurons of course exhibited direction-selective responses to the moving dot stimuli. But they also exhibited a remarkably simple dependence on the coherence of the motion: For a preferred direction of motion, MT responses increased linearly with increasing coherence; for anti-preferred direction motion, MT responses decreased linearly with coherence, although this decrease was quantitatively shallower than the increase associated with preferred directions. Furthermore, the temporal pattern of MT responses was relatively simple; after a fixed response latency and a brief onset transient, MT neurons responded briskly during stimulus presentation, exhibiting a generally flat firing rate throughout. Additional quantitative measurements yielded precise characterizations of the signal to noise of these sensory responses (Britten et al., 1993).

Recording from LIP during the dots task reflected an opportunity to observe the transformation of the precisely-characterized sensory signals in MT into a decision to move the eyes in LIP. Given that MT signals appeared to be relatively faithful and simple representations of the sensory stimulus, LIP responses had the potential to be approached as performing a computation upon the directional “evidence” coming from MT (Shadlen and Newsome, 2001). This relationship was supported by anatomical projections from the MT complex to LIP (Lewis and Van Essen, 2000a,b), as well latencies of direction- and coherence- dependent responses in LIP lagging those in MT during the dots task (Mazurek et al., 2003).

Single-unit recordings in LIP during the dots task revealed a pattern of response that still depended on motion direction and coherence, but that showed temporal dynamics substantially different from the simple MT responses. Instead of firing at a nearly constant rate that could be conceived of as an instantaneous representation of the sensory stimulus, LIP responses ramped upwards or downwards while the monkey discriminated the direction of motion (Shadlen and Newsome, 1996). It is this ramping of the LIP response during decision formation that has been interpreted as a neural correlate of the gradual accumulation of evidence during direction discrimination, for the purpose of ultimately making a saccade either into or out of the neuron's RF.

More precisely, the LIP spike rate would ramp up (or down) before an eventual saccade into (or out of) the RF, with a slope that depended systematically on the motion coherence. Higher coherences led to steeper ramps; lower coherences led to shallower ramps. It was as if the LIP firing rate was a direct neural instantiation of the accumulation of evidence (Shadlen and Newsome, 1996). Later in the trials, after the discrimination part of the trial was over, the neural response reached a common level immediately before a saccade into the RF. If the ramping responses during the moving dot stimulus reflected the accumulation of evidence, then the constant pre-saccadic level might be interpreted as a neural correlate of the results of that deliberation—perhaps a high or low state corresponding to the decision itself.

These initial interpretations of LIP activity were bolstered by later work that more rigorously focused on the decision-making phase of each trial. The initial LIP studies employed relatively long viewing durations and subsequent delay periods that were under the experimenter's control. Although this allowed the experimenters to distinguish the stimulus and the behavioral response by separating them in time, it was unclear exactly when the monkey made his or her decision. In fact, it was not just possible, but probable, that the direction discrimination task might be completed on most trials well before the stimulus was extinguished (Kiani et al., 2008). Later psychophysical results in both monkeys and humans confirmed this, suggesting that high coherence decisions were likely completed almost instantaneously (on order of 100 ms) but that lower coherence decisions reflected several hundreds of milliseconds of deliberation (Gold and Shadlen, 2003; Palmer et al., 2005).

In a critical neurophysiological study (Roitman and Shadlen, 2002), monkeys were trained to perform a response-time version of the dots task, in which they were allowed to make a saccade as soon as they desired. After training, the monkeys performed the task by indeed taking longer for lower coherences. Thus, just as their discrimination accuracy exhibited a systematic increase with coherence, their response times followed a systematic decrease along the same axis. During this version of the task, LIP responses ramped over approximately the same amounts of time that the monkeys were likely continuing to accumulate evidence. The LIP response at the time of the saccade was also striking: the coherence-dependent ramps converged within a few tens of milliseconds before the actual saccade. This was all the more consistent with the original supposition that LIP firing rates reflected not just the accumulation of evidence but also the end result of the decision process (of course, by allowing the saccade and the decision to ostensibly co-occur under the monkey's control, the interpretation of the neurophysiology requires additional care).

### Drift diffusion framework

The interpretation of coherence-dependent ramping of LIP responses as a neural correlate of the accumulation of evidence is not merely qualitative. In fact, LIP responses during the dots task are tempting to relate to a significant theory from mathematical psychology known as the drift-diffusion model. Originally posited by Ratcliff (1978)— and successfully applied to fit many findings in cognitive psychology (e.g., Ratcliff and Rouder, 1998, 2000; Ratcliff et al., 1999; Ratcliff, 2002)— the drift-diffusion model is derived from a quantitative analogy between the psychological accumulation of evidence to a decision bound, and the physical diffusion of a particle in the presence of absorbing boundaries. In the context of a perceptual discrimination task, the drift rate of this diffusion process can be controlled by the stimulus, in which stronger stimuli lead to more pronounced drift rates toward the corresponding bound. However, the process is noisy, so in the presence of weakly or moderately biased drift, there is variability both in which bound is hit, and the precise time at which it arrives.

The diffusion model thus makes predictions for the accuracy and speed of decisions using a single elegant mechanism whose heart is temporal integration. By conceiving of the process of accumulation as a noisy random walk of a decision variable toward one or another bound, a simple two-alternative task (like the dots task) could adopt the mathematical underpinnings developed by physicists to model diffusion processes. Although it required formidable insight to establish this conceptual relation, and considerable ingenuity to implement it, the psychological theories were ultimately able to rely on convenient mathematical expressions of bound-passing times that predict the speed and accuracy of decisions.

Despite the widespread application of this model to a variety of memory and decision-making tasks, its neurophysiological implementation did not receive much focus until recently (Ratcliff et al., 2003). Although there are certainly differences of opinion across the field, many cognitive psychologists likely remained agnostic about the underlying neural mechanisms. Just because a mathematical model based on noisy random walks often accounted for the pattern of reaction times, there was no consensus among researchers that the brain directly implemented such a process (although there was already remarkable progress relating neurophysiology to accumulator models of decision-making; see Hanes and Schall, 1996).

It was therefore rather striking how much LIP responses resembled the hypothetical processes in the drift-diffusion model over several hundreds of milliseconds. With the reasonable assumption that drift rate is a function of motion coherence, the well-known plots showing average LIP response as a function of time and coherence look a lot like the biased random walks of the drift-diffusion model. Furthermore, a very simple form of the drift-diffusion model does an excellent job of accounting for behavioral accuracy and response time in the moving dots task (Palmer et al., 2005). A simulation of LIP responses confirmed that they are well-approximated by an underlying temporal integration of noisy sensory signals from area MT (Mazurek et al., 2003), although a more realistic model using real LIP responses has yet to be undertaken (but see Purcell et al. (2010) for a successful implementation of this approach in the frontal eye fields).

To summarize, LIP responses during the dots task resemble the variables posited by the drift-diffusion model, and the drift-diffusion model accounts for psychophysical performance in the same task. This led many to adopt a framework in which LIP activity was a direct neural instantiation of the decision-making process described by drift-diffusion, i.e., that the accumulation of evidence as described by drift-diffusion was explicitly represented in the spike counts of single neurons in LIP. Because drift-diffusion has a clear mathematical implementation, the fidelity with which LIP matched this process makes it a particularly appealing quantitative form of a “neural correlate.” In the following section, we review some extensions of this work that further generalized this quantitative link and made the correlation between LIP and a diffusion process even more striking.

### Extensions of the dots task

This basic link between LIP response and a theoretical decision variable has been aggressively explored and extended over the last decade or so. For example, Churchland and colleagues (2008) included a condition with four choice targets (and four potential coherent motion directions) instead of the conventional two. They observed many of the same aspects of LIP responses described above (e.g., coherence-dependent ramping), but also observed a lower initial firing rate in the four-choice trials. This was interpreted as a lower starting point for evidence accumulation, which is intuitive, because with more alternatives the decision will likely require more deliberation. A 3-alternative version of the dots task has also received psychophysical and modeling attention (Niwa and Ditterich, 2008; Ditterich, 2010). Furthermore, similar effects of the number of choice alternatives have been observed in other tasks in LIP (Louie et al., 2011) and other oculomotor areas (Basso and Wurtz, 1998; Lee and Keller, 2008).

In another ambitious extension, the dots task was modified to contain a third target option, presented at end of moving dot stimulus that constituted a “sure thing”— a small but certain reward (Kiani and Shadlen, 2009). On trials in which the monkey eventually chose this smaller but certain stimulus, the LIP response during the dots was muted. If LIP responses reflect the accumulation of evidence, a slightly lower level would suggest trials in which evidence was not acquired as quickly as usual. This lower level of accumulated evidence could in turn correspond to a lower confidence, and hence the selection of the “sure thing” target on those choices.

Another interesting task variant generalized the dots task to less certain mappings between motion direction and saccade generation (Bennur and Gold, 2011). In this version, the two choice targets were colored differently, and the monkeys learned that a particular direction of motion corresponded to choosing a particular *color* target (as opposed to the usual spatial selection of a target in a particular location that is consistent with the direction of motion). Critically, the differential target colors were revealed either at either an early, middle, or late period of the task. Although there are many nuances in the results of this study, the core result was that the conventional decision-related signals emerged in LIP when the choice targets (and hence the direction of the saccade) were disambiguated. That said, some neurons showed decision-related activity before that disambiguation, although of course the mapping between saccade direction and this activity was idiosyncratic. The interpretation, couched in the context of the drift-diffusion model, is that these latter LIP neurons perform a more general, and response-independent accumulation of evidence (Fanini and Assad, 2009), complementing the more conventional sensorimotor mapping seen in the usual version of the task.

Finally, a trio of recent studies has explored how other decision-related factors are reflected in LIP during the performance of the dots task. One study (Rorie et al., 2010) manipulated the reward associated with different directions of motion, and observed that LIP responses were higher for the direction with the larger reward. Because this reward effect was present from early in the trial, and was roughly additive in nature, these physiological observations can be interpreted as the reward affecting the starting point of the evidence accumulation, without much affecting the rate of the accumulation of evidence. Another study (Hanks et al., 2011) manipulated the relative prior probabilities of the two directions of motion, and found that LIP spike rates were larger for the more likely direction, but that the magnitude of this increase depended on stimulus reliability (and/or elapsed time). These observations lead the investigators to posit a novel modification to the drift diffusion model, where elapsed time is used to determine how much weight to apply to sensory evidence relative to prior probabilities. In contrast to those findings, another study manipulated prior probabilities (Rao et al., 2012) but found a largely additive effect on LIP responses instead. Such a modulation can of course be interpreted in terms of an additive offset of the accumulation of evidence, although it differs from the dynamic bias signal observed by Hanks and colleagues. The reason for these different effects of bias may be due to experimental differences (i.e., the latter study used an explicit visual cue to signal changes in prior probabilities from trial to trial), but the only definitive point that can be made is that both types of effects could be interpreted in terms of simple effects on a drift diffusion process. Other studies using different oculomotor choice paradigms have also observed strong modulations of LIP response for these non-sensory components of decisions (e.g., Platt and Glimcher, 1999).

These examples suggest that the link between LIP and the drift diffusion model is robust and general. In these novel variants and extensions of the task, LIP responses can still be interpreted as directly mapping on to the accumulation of evidence over time, up to (or near) the point of making a decision. The goal of the following sections, however, is to contemplate phenomena and levels of analysis that fall outside of this neural correlate framework in the hopes of gleaning additional insight into LIP's function.

## Empirical questions

The similarity between LIP responses during the dots task and the accumulation of evidence modeled by the drift-diffusion framework is certainly appealing. It reveals a quantitative, parametric relation between spike rates in LIP and an inferred decision variable, across multiple variants of the dots task. However, looking beyond these successes reveals a number of empirical questions (still accessible within the dots task) that are yet to be systematically investigated. When acknowledging the richness of sensorimotor responses in LIP, it is not surprising that there are many nuances of response that might provide leverage into the computations performed in this area.

Although it seems trivial (and less interesting) compared to LIP's ramping response, the largest response seen in many LIP neurons is elicited simply by the appearance of choice targets at the start of the trial. The onset of the choice target within the RF can create a quick and robust response, as can also be seen in simpler instructed-saccade tasks (Bisley et al., 2004). In the context of the dots task, this strong transient response is typically considered irrelevant because it occurs well before the onset of the moving dots and the decision phase of the trial. This response is sometimes not evident in published peri-stimulus time histograms that align the responses to the onset of the moving dots (e.g., Shadlen and Newsome, 2001; Huk and Shadlen, 2005, but see Churchland et al., 2008). Likewise, it should also be noted that the classical coherence-dependent ramping during dots viewing is sometimes very modest relative to the overall response range of the neurons (Kiani et al., 2008; Rorie et al., 2010; Rao et al., 2012) and can exhibit idiosyncrasies (Roitman and Shadlen, 2002).

Given the large magnitude and unknown time course of this decision-irrelevant component of the response, it is important to characterize how it interacts with decision-related activity. The most obvious test would be to simply withhold presentation of the choice targets until after the moving dots. If the targets always occurred in stereotyped location, this manipulation would not exert a significant effect on behavior. However, it is far less obvious what would happen to the response dynamics during the moving dot stimulus and decision formation. If LIP really reflected a drift diffusion process (such that the spike rate mapped on to the accumulation of evidence in a fixed manner), then the LIP response should be insensitive to this manipulation, and increase to the same level as it does in a normal trial.

Alternatively, the usual levels of LIP response seen in the dots task might reflect the summed contributions of visual drive and decision-related activity. If that were the case, LIP responses might start from a considerably lower level than is commonly observed. Although it would obviously be interesting to see what happened to the downward ramps (ones associated with choices of the target outside the RF) given that they might approach zero spikes/sec, it would be perhaps more important to evaluate whether the upward ramps (associated with choices of the target in the RF) were affected by this manipulation. Other possibilities abound (e.g., an extreme example would be that the visual target gates decision-related activity through LIP)—but the key point here is simply that we know very little about some rather basic components of the sensorimotor processes reflected in LIP. If LIP implements an unwavering neural correlate of a drift-diffusion process underlying decision formation, its responses should be impressively robust to manipulations of decision-irrelevant factors that are known to exert large effects on LIP spike rates. Given that some experiments discussed above have already extended decision-related aspects of the task (i.e., the number of choice alternatives) by (necessarily) changing the visual stimulus geometry, it would be helpful to have a general analysis scheme that could parcel out the purely sensory effects of these manipulations from the changes in decision processes of interest (although some of these studies have attempted to address this issue with clever control conditions).

Another standing question has to do with the early phase of the LIP response before the ramping responses start. After the onset of the moving dots, there is an approximately 200 milliseconds-long period in which responses do not depend on motion direction or coherence, and instead undergo a roughly stereotyped dip and rise. This phase has been interpreted in many different ways—e.g., as a reset of a neural integrator (Sato and Schall, 2001; Roitman and Shadlen, 2002; Mazurek et al., 2003; Huk and Shadlen, 2005), or as a sensory or attentional interaction between the choice targets and the onset of the moving dots (Ben Hamed and Duhamel, 2002; Wong et al., 2007). Although these intriguing propositions exist, this phase of the response has received little direct experimental effort. One thing we do know is that this phase is better thought of as a latency of LIP relative to the dots, as opposed to a period of time in which the ongoing moving dot stimulus is ignored (behaviorally and neurally). The clearest evidence that this early motion matters comes from experiments that manipulated the time course of motion coherence: changes in the motion signal that occur while LIP is undergoing the dip-and-rise still affect neural responses (as well as psychophysical performance) with the appropriate 200 ms latency (Huk and Shadlen, 2005; Kiani et al., 2008). Moreover, monkeys can still perform the task above chance for very brief presentations of the dots (Gold and Shadlen, 2000, 2003).

There are some simple experiments that could shed light on the computational meaning of the dip-and-rise. If this pattern is due to a “reset,” then performing a version of the dots task in which monkeys are trained to “start over” their integration later during the moving dots should create new dip-and-rises accordingly (Bennur and Gold, 2011). If this pattern is instead due to an attentional shift from the targets to the onset of the moving dots, a cue that systematically precedes the moving dots should temper or modulate the dip and rise. Likewise, if the interaction between targets and dots is more of a passive visual interaction, then simple manipulations of the relative intensities of the two types of stimuli (e.g., size, contrast) should reveal such wide-field interactions. Although these are straightforward experiments in nature, they are interesting to contemplate simply because they emphasize that we do not understand the significance of the first 200 ms of LIP response during the formation of decisions. This seems in part because the drift-diffusion framework does not naturally offer up an interpretation, other than to suggest that LIP reflects drift-diffusion with a particular latency.

In summary, there are many unanswered empirical questions within the dots task paradigm. These are rather basic questions that focus on how simple visual elements of the task drive LIP and interact with the decision-related activity. Although these may sound less lofty than the interactions between multiple cognitive factors of the sort that are currently receiving attention, we argue that understanding the basic visual components of the task is not just a tractable exercise for characterizing basic sensory computations in LIP, but a critical underpinning for more precise interpretations of the other, less-well-understood (but perhaps more intriguing) cognitive signals seen in LIP.

## Implementation issues

LIP receives so much attention primarily because the temporal dynamics of its responses span sensory, cognitive, and motor functions. Classically, many neurons in LIP are known to exhibit strong persistent activity during memory-guided saccades. When a future saccade target flashes on the screen within the RF of an LIP neuron, the neuron responds strongly; and when the monkey eventually saccades to the remembered target location, the neuron also responds strongly. But what is more impressive is that these same LIP neurons also exhibit temporally-persistent activity that bridges the delay period between the target's flash and the memory-guided saccade.

The temporal dynamics of LIP responses during the moving-dot direction-discrimination task also suggest an important role in bridging sensory and motor functions. As described earlier, LIP responses ramp upwards or downwards over time, in a choice- and coherence- dependent manner that is consistent with the accumulation of evidence over time. Such dependencies were initially observed in “fixed-duration” versions of the task in which the experimenter presented the stimulus on every trial for a known amount of time (1–2 s) (Shadlen and Newsome, 1996, 2001). Although this was already an intriguing result, the temporal dynamics of the responses were difficult to interpret precisely, because it was not known exactly when a decision was made (and presumably, when the accumulation of evidence stopped). Therefore, later work using a free-response (“response time”) version of the task yielded temporal dynamics that appeared to even more neatly line up with the accumulation of evidence leading up to a decision about motion direction (and hence to move the eyes to a particular choice target) (Roitman and Shadlen, 2002).

To test the hypothesis that spike rates in LIP reflected the temporal integration of evidence related to decision formation, a pair of studies injected brief “motion pulses” into the standard moving-dots stimulus (Huk and Shadlen, 2005; Kiani et al., 2008). These brief events serve both as a way to create a time-varying stimulus that should yield a specific change in the temporal dynamics of LIP, as well as being temporal “tags” that help disambiguate the timing of LIP responses relative to stimulus events. In the original study (Huk and Shadlen, 2005), motion pulses in either the same or opposite direction of the dots made LIP responses increase or decrease in a direction-dependent manner. Furthermore, these perturbations persisted in the LIP response for several hundred milliseconds. This was a critical result, as it provided the first direct evidence that LIP firing rate at a particular point in time was a function, not just of the current stimulus, but of the previous stimulus history (within a behaviorally relevant time frame). In other words, LIP firing rates approximated the time-integral of relevant sensory data during decision formation, and “remembered” the motion pulse. A second study (Kiani et al., 2008) extended this basic result and more quantitatively probed how these pulse effects might change over time under the assumption that evidence was not accumulated forever, but just until enough was attained to make a decision.

Although these studies serve as rigorous engineering-style assays of the time-integration properties of LIP, they shed very little light on *how* neurons might perform such temporal integration. At first glance, there appear to be two extremes of explanation: either cells are individually endowed with intrinsic biophysical mechanisms that allow them to continue responding to inputs that are no longer present, or they are situated in a circuit that creates persistent activity by virtue of its network architecture. In fact, the extremes of this dichotomy are not the only possibilities worth considering, as theoretical work has shown that both slow intrinsic time constants *and* recurrent network connectivity are likely necessary to support persistent activity that is relatively stable over appropriate timescales (Tegnér et al., 2002; Wang, 2002).

Because the long temporal integration of LIP neurons is a rather unique property compared to the more “real time” response dynamics of basic sensory and motor neurons, we propose that temporal integration *per se* deserves at least two lines of focus. First, is the temporal integration capacity of LIP neurons fixed (by virtue of the intrinsic and extrinsic factors described above), or can it vary? Second, do LIP neurons compute this time integration, or do they receive signals that are already time-integrated? A variety of experiments discussed below could answer these questions. Loosely, these can be divided into “single neuron” issues and “network” issues.

### Single neuron mechanisms

There is already a tacit assumption that the temporal integration capacity of LIP neurons is somewhat fixed. In the context of the moving dots task, experimenters typically use the observance of persistent activity not just as a general tool for confirming that their electrode is in LIP, but also as a cell selection criterion (Shadlen and Newsome, 1996) for gathering data from neurons that will show ramping temporal integration. However, even within this selected subpopulation of LIP neurons with persistent activity, response heterogeneity is significant (Premereur et al., 2011), and many neurons exhibit weak or idiosyncratic forms of temporal dynamics that do not suggest robust or canonical temporal integration.

The application of a “robust persistent activity” criterion for choosing whether or not to perform an experiment while recording from that particular neuron reflects a strong assumption that certain LIP neurons are robust time-integrators, while others are not. By then presenting the average activity of the subset of LIP neurons with strong persistent activity as a “population response” that is a quantitative neural correlate of a decision process, it also reflects the assumption that the signals in these cells can somehow be distinguished from other signals in LIP in forming the decision. These are strong assumptions.

There are several potential ways to gain insight on these issues. First, if the temporal integration properties of cells are relatively fixed, the degree of temporal integration seen across tasks should be stable, as it would derive from an intrinsic cellular mechanism (considered in Durstewitz and Seamans, 2006). For example, if a cell exhibited robust persistent activity during memory-guided saccades, it should exhibit strongly linear ramping during the dots task. On the other hand, cells that show decaying persistent activity during memory-guided saccades might exhibit dots-task responses that saturate. In the simplest case, the decay of persistent activity could be fit with an exponential, and the value of this time constant of decay would explain the time constant of saturation in the dots-task responses. In relevant work from a visual search paradigm, NMDA receptors (which have a distinctively long time course) have been implicated in neural temporal integration (Shen et al., 2010; see also Standage and Paré (2011) for associated modeling). It is likely that cellular mechanisms such as NMDA receptors are critical within a recurrent network architecture (Wang, 2002).

Of course, it remains to be seen whether simple characterizations of temporal integration properties are even appropriate, but the general approach holds regardless of the specific functional form needed to fit real data. Primarily, it remains to be seen whether the persistent-activity criterion is even justified. Although there are likely anecdotes and expert hunches underlying this assumption, systematic direct tests of this assumption are currently absent from the literature. The reason for this might be that one would need to record from neurons without strong persistent activity to see if they indeed did not carry decision-related activity during the dots task. Although researchers (especially ones that use animal models) are wisely cautious of performing experiments in which they expect not to see an interesting response, these measurements are a necessary part of understanding the neural computations performed by LIP neurons. It is likely that such measurements would also provide additional insights into the variety of signals carried by “non-canonical” LIP neurons, of which there are many.

This last point may be imperative for forward progress. Our understanding of early visual areas like V1 has culminated in a characterization of different cell types, which has in turn suggested distinct neural computations and even a potential hierarchy (e.g., from simple to complex cells). Despite the large amount of work in LIP, we are not close to such a nuanced answer. Although it is known that cells in LIP exhibit varying degrees of visual, memory, and motor responses (Barash et al., 1991), considerably more emphasis could be placed on understanding the single neuron computations. The vast majority of work in the dots task has focused on plots of population response, or in cell-by-cell analyses that use derived variables extracted to test a very limited hypothesis. This contrasts even with work on a related oculomotor area, the frontal eye fields, for which the appreciation and categorization of cell diversity has been a long-standing element (Bruce and Goldberg, 1985; Cohen et al., 2008).

Beyond the need for continued progress in appreciating different cell types (Premereur et al., 2011), there is relatively little fine-scale understanding of the architecture of LIP. It has been subdivided based on anatomy and connectivity into dorsal and ventral components (Lewis and Van Essen, 2000b), and one study has suggested a more “cognitive” role for neurons in ventral LIP (Liu et al., 2010). And although dots-task studies have gradually emphasized (and even targeted) LIPv, there is again very little published data that test whether decision-related signals are indeed represented preferentially in a distinct group of cells or location. This is another thorny issue to address in practice, given that with conventional single-electrode/single-neuron techniques (coupled with a dorsal-to-ventral penetration trajectory), the simple probability of encountering a desirable neuron grows over time, and in this case depth. Multi-electrode or stacked-array recordings might provide greater leverage on this issue. Some investigations of LIP cell types and circuitry have been performed using other techniques (Lynch et al., 1985; Blatt et al., 1990; Schall et al., 1995; Ferraina et al., 2002; Bakola et al., 2006), but significant progress at fine spatial and computational scales remains to be made. And analogous work in other animal models will be an important complement, given the array of powerful tools at the disposal of researchers using smaller animals (e.g., Atallah et al., 2012; Raposo et al., 2012).

### Network

Other implementation questions are more network-oriented. Perhaps the most glaring shortcoming in our understanding is the lack of quantified inter-neuronal correlations. The vast majority of analyses have focused on linking LIP activity *on average* with corresponding aspects of behavior. However, quantities related to the average spike rate (say, averaged over neurons, or repetitions of certain types of trials) can obscure the dynamics within the population on single trials. One bit of leverage in previous papers has involved correlating the LIP response on single trials with the reaction time of the monkey, which has often indicated a significant negative correlation (i.e., stronger responses are correlated with faster RTs; e.g., Roitman and Shadlen, 2002). However, a more direct attack will of course involve the measurement of multiple neurons simultaneously. An important first step has very recently been published that demonstrates the utility of these measurements (Bollimunta et al., 2012). Such measurements will provide a more thorough estimate of the population response within LIP on single trials (in fact, undifferentiated multi-unit “hash” may be a particularly powerful metric in this domain, although this suggestion is admittedly in tension with the prior section's emphasis on understanding single unit computations). Recent work focused in another posterior parietal region (the parietal reach region, PRR) has demonstrated the utility of moving beyond single-unit spike counts (Pesaran et al., 2002; Hwang and Andersen, 2009, 2010, 2011, 2012), as well as one study that gained leverage from distinct signals seen in local field potentials in LIP during the dots task (Bollimunta and Ditterich, 2012).

Multiple-neuron recordings also allow for the quantification of inter-neuronal correlations. Although correlation is always an important factor in understanding the amount of information that can be signaled by a neural population, it is a particularly valuable piece of information in understanding the mechanisms underlying temporal integration in LIP. Theoretical models of LIP based on recurrent connectivity (resulting in attractor dynamics) should make rather distinctive predictions for the magnitudes and time courses of neuronal correlation (Wang, 2002; Wong et al., 2007). Although initial models of LIP have assumed a fixed correlation extrapolated from measurements in sensory areas, attractor dynamics would likely be manifested in a transition from relatively weak correlations to very strong correlations at the time of decision formation.

It is also not known whether such relations are fixed properties of the network, or whether they themselves are dynamic, depending on the nature of the task. For example, if two neurons with partially-overlapping RFs contain a shared choice target, they should function as part of the same assembly; if the task is then changed so that those same two neurons now now contain different choice targets in the non-overlapping portions of their RFs, they should now participate in competing pools (Bollimunta et al., 2012). Whether their responses and inter-neuronal correlations are fixed, or depend on such task changes, will provide important insights into the flexibility of the circuitry. In general, simultaneous multi-neuron recordings are needed for furthering our understanding of the network mechanisms in LIP. Such experiments are just starting to be reported (Bollimunta et al., 2012), and more results from this enterprise are eagerly anticipated. Similarly-minded studies have already identified context-dependent responses in related brain areas, such as MT (Cohen and Newsome, 2008).

Another more general issue that deserves more work is where LIP is situated in the decision-making circuit. Anatomical evidence provides little constraint on the circuitry, instead revealing a pattern of promiscuous, bi-directional connections between many parts of posterior parietal “association cortex” and a variety of sensory and oculomotor brain regions. An intriguing bit of physiology that should receive more attention is the pattern of latencies across brain areas. LIP itself exhibits a relatively long latency: After a 200–225 ms dip-and-rise phase that does not depend on stimulus or predict the eventual behavioral response, LIP exhibits its customary ramping activity. This is a very significant latency relative even to MT, which responds to simple visual stimuli with a lag on order of ∼80 ms (Britten et al., 1993; Raiguel et al., 1999). Thus, LIP's decision-related activity, although postulated to reflect the time-integral of relevant directional input from MT, lags behind the MT signals by at least 120 ms. So, based on simple latencies, we should assume that the circuit distance from MT to LIP is one and a half times as far as the distance from the retina to MT. Of course, assigning latencies to LIP is a somewhat dubious exercise, given that the form of its response does not have as distinct an onset as a purely sensory response. Regardless, such a ballpark analysis suggests that a variety of neural computations (and synapses across brain areas) could lie between MT and LIP. One caveat is that the latencies of other signals in MT and LIP may not follow such a simple temporal relation (Saalmann et al., 2007; Herrington and Assad, 2010).

A number of experiments have focused on recording single-neuron responses during the dots task in other oculomotor brain areas, with recent emphasis by Gold and colleagues. In short, recordings from superior colliculus, caudate, and FEF all reveal decision-related ramping responses (Horwitz and Newsome, 2001; Ding and Gold, 2010, 2011), suggesting that the signature aspects of LIP activity during the dots task may be the consequence of a distributed computation (or the widely-disseminated results of a computation). It is likely that subtleties in the relative latencies, statistical relations to behavioral variability, and qualitative effects beyond the ramping component will ultimately inform a circuit-level understanding of decisions in the dots task. For the time being, it appears that collecting more information about the responses of multiple areas, preferably under identical task conditions (and training histories) will be necessary. Comparisons between parietal and prefrontal activity have indeed begun to yield insights into working memory and oculomotor behavior (Qi et al., 2010; Katsuki and Constantinidis, 2012).

### Summary

This discussion reveals that the relation between LIP and the accumulation of evidence is primarily a descriptive link: one mimics the other with good fidelity under some conditions. However, we know precious little about how LIP neurons might come to reflect such temporal integration. There are both single-neuron and network measurements that are now feasible and which could begin to unpack the neural computations that underlie LIP's neural correlates of decision formation. Although continued demonstrations of such correlations in new extensions and varieties of decision-making tasks provide an important phenomenological catalog, we suggest that neurophysiology can now be the appropriate tool for identifying how such signals arise in LIP, given that these signals appear to be a crucial and basic component of the transition from sensory processing to cognition. These measurements will benefit from having a common analytical framework for extracting components of the responses and quantifying factors such as latencies.

## Ecological constraints

From an experimentalist's perspective, one of the most appealing aspects of the moving-dots task is that it requires hundreds of milliseconds of psychophysical deliberation. This is a long period of time to concurrently measure neural responses, allowing for insights into the time course of decision formation. Given that most visual tasks require only short (<100 ms) of temporal integration, the quarter- to half-second (or more) of decision formation time during the dots task is precious.

However, the long time course of this task raises the specter of ecological relevance. A typical trial in this task involves a few hundred milliseconds of stable fixation, a few hundred milliseconds associated with the onset of the choice targets, several hundred milliseconds of the moving dots stimulus, and sometimes a post-stimulus delay period, before the ultimate saccadic response. A trial, from start to finish, can rarely be completed in less than a second. This pacing contrasts starkly with natural oculomotor behavior, in which saccades can occur on order of 3–5 times per second (Findlay and Gilchrist, 2003).

Raising this issue is not meant as a criticism of artificial stimuli and well-controlled experiments (Rust and Movshon, 2005). However, it may not be correct to draw a full analogy between the use of bars and gratings and dots to understand sensory processing, and the use of arbitrary tasks to probe the mechanisms of cognition. Presuming that LIP also functions outside of the laboratory, it probably evolved as part of a circuit that guides saccadic and attentional exploration of visual scenes (indeed, it exhibits interpretable response patterns during relatively unconstrained oculomotor behaviors; e.g., Ipata et al., 2006). If the natural neural computations in this area guide a saccade every 200–300 ms, what do the responses of LIP neurons tell us when the monkey must maintain stable fixation (i.e., avoid doing what they would naturally do) for approximately an order of magnitude longer? (Relatedly, little is known about whether the nature of these saccades differentially affects LIP, i.e., conventional saccades related to visual exploration, versus microsaccades).

Of course, this discussion cannot provide a definitive answer to whether the unnatural timing of saccades in the dots task can still reveal basics of function, but this point is worth keeping in mind for at least two reasons. The first is as a reminder that some of the signals inferred from LIP activity might reflect the circuit being inhibited from its natural function (for example, the timing and urgency signals posited in recent work (Churchland et al., 2008; Hanks et al., 2011) might be an inevitable consequence of the circuit “gearing up” for the next eye-movement after an unnatural period of inhibiting such behaviors). Second, this tension between experimental and natural time scales of oculomotor behavior suggests a variety of intriguing experiments that may shed light upon how to interpret responses in LIP.

If saccades typically occur several times a second, but interesting cognitive decisions require deliberation over longer periods, it is unclear what the decision-related signals seen in LIP during the dots task tell us about the general neural computations underlying the accumulation of evidence. Perhaps we are simply studying the “tail of the distribution”: the mechanisms that underlie the rare moments in which primates cannot move their eyes for a second or more, but need to be planning the next eye movement (as in the case of truly “covert” attention). Relatedly, we may simply be pushing the circuit to reveal its capabilities, regardless of its modal functional time scale. However, the more exciting possibility raised by this topic is simply that LIP may carry decision-related signals that are dissociable from eye-movements.

The possibility of divorcing decision-related signals from oculomotor behavior has been raised by the results of Bennur and Gold (2011), who found that some neurons carried decision signals before an eye movement could be planned (before the mapping between moving dot direction and saccade target location was revealed). Likewise, in a task that replaced the moving dots with symbolic probabilistic cues, Yang and Shadlen (2007) showed evidence-related “steps” in LIP firing levels during the sequential presentation of stimuli (far in advance of an eye movement) that had particular log-likelihoods of reward associated with them. Other results in the literature also point in this direction, as a variety of categorization task experiments have revealed selective LIP responses that cannot be easily interpreted in terms of saccade planning (Freedman and Assad, 2009, 2011). Of course, there is also a long literature attempting to dissociate saccade intention signals from spatial attention. Also, LIP RFs exhibit anticipatory remapping, such that neurons will respond not just to a stimulus in the RF, but also to a stimulus that will be in the RF after the impending saccade (Duhamel et al., 1992). Finally, a variety of saccade metrics are not tightly coupled with LIP spike rates (e.g., Platt and Glimcher, 1999; Pesaran et al., 2002; Dorris and Glimcher, 2004; Bendiksby and Platt, 2006), even during the dots task (e.g., Shadlen and Newsome, 2001).

In summary, there is no doubt that tasks involving oculomotor responses are an effective means for eliciting strong and spatially-selective responses from LIP. Simultaneously there is a growing body of evidence suggesting that LIP can carry decision-related signals that are not tightly coupled with the plan to make a saccade into or away from the RF. However, we currently have very little leverage on understanding whether the slow ramping activity seen during the dots task—perhaps the most-studied “decision signal” in LIP—can be dissociated from the plan to make a particular saccade. Basic experiments are easy to envision, and seem particularly motivated in light of recent exciting developments that have posited a tight link between decision signals during the dots task and the recruitment of particular effectors (Resulaj et al., 2009; Selen et al., 2012). However, such experiments will entertain time scales that are shorter (e.g., natural fixation distributions) and longer (e.g., estimations of reward rates) than are commonly considered in conventional “trials,” and so (just as in the prior sections) these computational questions call for an analysis approach that is general enough to model the relation between a wide array of external variables and LIP responses.

## Conclusions

This discussion began by describing the face-level similarity between LIP activity during the formation of decisions in a random-dot direction discrimination task, and the psychological process of evidence accumulation hypothesized to underlie those decisions. In a quantitative sense, the average LIP response over time bore an uncanny resemblance to the sort of noisy accumulation process posited in models within the drift-diffusion framework. Since the original reports of such a “neural correlate” of decision formation in LIP (Shadlen and Newsome, 1996, 2001; Roitman and Shadlen, 2002), further work within this experimental paradigm has built a large body of correlational phenomena linking LIP physiology and the formation of decisions in the context of a drift diffusion model—and has gone on to begin using the physiology to refine and extend the classical psychological models (see Gold and Shadlen, 2007; Wong and Huk, 2008; and Churchland and Ditterich, 2012 for more comprehensive reviews).

Although this is a remarkably rigorous neural correlate, we have attempted to identify several holes in our understanding of what LIP responses mean. For example, in the empirical domain, we pointed out that it is not yet known whether LIP responses are an invariant and pure neural correlate of the accumulation of evidence, or rather whether they carry a decision-related signal that can be mixed with other (sensory and motor) signals. If the latter is true, then we must contemplate whether downstream structures can properly de-multiplex the LIP response in order to distinguish the decision signal from extraneous factors that also elicit spikes in LIP. In terms of implementation, we also noted that very little is known about how LIP responses might come to reflect the time-integral of relevant sensory evidence: is it a remarkable intrinsic property of these cells or more of a distributed network computation? Finally, we questioned the ecology of the dots task, raising the question of what the task might tell us about decision formation over time, given that it involves stable fixation for roughly an order of magnitude longer than natural oculomotor behavior involves.

In summary, there are a large number of unanswered questions, and although they fall under a wide array of rubrics (summarized above), they are all fundamentally about what and how LIP neurons compute; i.e., characterizations of the relevant inputs, the corresponding outputs, and the basic principles that predict the outputs from inputs. The answers to these computational questions are critical for understanding what LIP does, and should also provide important links to other studies of LIP during tasks focused on shifts of attention, eye-movement planning, visual search, categorization, valuation, and other phenomena. We suggest that continued demonstrations of neural correlates of a decision variable in LIP will not answer these questions. Instead, a new analytic perspective may facilitate work that emphasizes neural computations over neural correlates. In the next section, we propose an “encoding-decoding” framework and explain why our current understanding of LIP is at a critical stage that requires it.

### The encoding-decoding framework for LIP

Although LIP is intriguing because it so often appears to carry signals that are distinct from “simple” sensory and motor processes, this does not mean that the analysis of LIP responses requires novel machinery. In fact, LIP's apparent complexity may be easiest to crack if we adopt an analysis strategy that starts with an explicit focus on the observable sensory and motor elements. This leads us to what we call an “encoding-decoding” framework.

The first part, *encoding*, involves building a descriptive model of if and when an LIP neuron will fire an action potential, given various external variables. Note that although the term “encoding” is usually applied in this context to describe the role of sensory neurons, here we mean it in the more generic sense of modeling a neural response given external variables. In the case of LIP, and the tasks used to study it, there is a long list of potential factors. In even a simple version of the dots task, there are several stimuli that could drive LIP: the fixation point, the choice targets, and the moving dots. Furthermore, encoding models are not constrained to be causal, so one can also contemplate task elements that might be preceded by LIP responses, such as buildup activity preceding the saccadic eye-movement. Finally, they can be easily extended to consider factors that are outside core analyses of the dots task, but which other lines of work have suggested are important in LIP, such as rewards (or lack thereof), and the recent history of behavioral responses (or of trial outcomes). Along these lines, one underappreciated study showed the utility of this approach by decomposing LIP responses into basic three components: sensory, motor, and “cognitive” (Ipata et al., 2009). We suggest that this sort of approach can be vastly expanded and generalized within a principled statistical framework.

Of course, implementation of such a general encoding model will be nontrivial, and would require both judicious experimental design and an appropriate means for both separating the effects of all these events as well as combining them to generate a single output (spikes). Work in other systems has relied on a generalized linear model (Simoncelli et al., 2004; Truccolo et al., 2005), which involves a front end of linear filters followed by a conventional nonlinearity and a probabilistic spike generation step. Although such encoding models have been primarily applied to earlier sensory or later motor regions, their flexibility may make application to sensorimotor areas like LIP especially illuminating. In short, they would allow for letting the data (and a careful record of all potentially-relevant events) tell us what makes an LIP neuron spike, within a framework that assumes the multiple factors combine straightforwardly (e.g., obey superposition).

Regardless of implementation, a successful encoding model could yield significant insights into the neural computations performed by LIP. To start, one could ask to what degree the response of LIP is a function of the multitude of events going on in even the simplest tasks. Furthermore, each component driving the LIP response could be isolated. This would allow further analyses to focus on a particular component of interest, such as the response to the dots, as isolated from potential responses to the target and related to the impending saccade. Distinguishing these components might shed light upon the significant heterogeneity seen across LIP neurons (e.g., Barash et al., 1991; Premereur et al., 2011). Finally, another potential benefit of such an encoding decomposition would be comparisons across studies that use very different tasks: The elements that are typically shared across tasks could be distilled out (such as responses to targets and saccades), so that the remaining distinct response components could then be interpreted and compared. Ideally, an encoding model would serve as a common language for understanding which signals are present in LIP across a variety of tasks and studies—and perhaps for resolving apparent differences based on subtler differences in seemingly trivial elements, such as the timing or locations of visual stimuli (e.g., the choice targets).

The other side of this framework, *decoding*, would involve taking LIP responses and trying to infer the presence or value of some external variable. Again, for clarity, although the term “decoding” is often used in this context to describe what LIP is thought to do, here we mean it more generally, as in attempting to estimate an external event given a particular neural response. This is an important complement to the encoding perspective, especially when a brain area potentially responds to a multitude of factors in the task. For neurons that only respond to one component in a stimulus, decoding the value of that stimulus is a relatively simple complement to encoding which provides insight into the noisiness and fidelity of the representation of that feature. But for neurons whose output is the superposition of multiple factors, decoding the value of a single variable is a richer puzzle. It requires the decoder to grapple with de-multiplexing a complex neural response, and hence allows for assessment of how robust and invariant a particular neural signal is in the face of other factors also driving the neuron.

A decoding analysis in LIP will benefit from (or even require) a successful model of encoding. If the multiple signals and computations performed by (and reflected in) LIP can be accurately identified from the encoding perspective, then decoding algorithms can attempt to extract these components. The performance of such decoding efforts would allow for quantitative probing of the relation between LIP and various sensory and motor functions. For example, one could ask, within a common quantitative framework, the degree to which LIP responses reflect the direction of motion in the stimulus, versus the degree to which they reflect the decision about the direction (i.e., the saccade). In addition to establishing a common ground for such quantitative assays, an explicit focus on decoding would motivate consideration of how LIP itself might be “read out” along the oculomotor pathway (see also Mirpour and Bisley, 2012). If the instantaneous spike rate within LIP really does directly map on to a decision variable, subsequent stages would simply need to integrate LIP responses over a brief window to estimate that rate. On the other hand, alternate (i.e., longer, and time-varying) weighting schemes might extract more information from the spike train, meaning that LIP responses would not necessarily reflect the final (or optimal) decision variable, but rather a partial sensorimotor transformation. Although these possibilities raise more questions than they answer, the value of decoding as distinct from encoding has already been appreciated in LIP: Recent work has begun to use simple decoding metrics as a way to test between different functional theories of LIP (Quian Quiroga et al., 2006).

In summary, the *encoding-decoding framework* that we make explicit here is simply an application of an already-mature approach for the study of sensory and motor function. It provides an interpretive structure that should guide experiments and analyses, but is inherently data-driven in what it reveals. It also formalizes an arena for the exchange and comparison of data across multiple studies and laboratories. The extension of this framework from sensory and motor function to that of sensorimotor integration may be especially challenging, but equally enlightening.

### Conflict of interest statement

The authors declare that the research was conducted in the absence of any commercial or financial relationships that could be construed as a potential conflict of interest.
